# Big Five personality and mind wandering in athletes: mediating role of trait anxiety

**DOI:** 10.3389/fpsyg.2024.1232312

**Published:** 2024-02-13

**Authors:** Yueming Li, Jifei Ma, Yue Xi, Jieling Li

**Affiliations:** ^1^School of Physical Education, Hebei Normal University, Shijiazhuang, China; ^2^Department of Physical Education, Cangzhou Normal University, Cangzhou, China; ^3^Key Laboratory of Measurement and Evaluation in Exercise Bioinformation of Hebei Province, Shijiazhuang, China; ^4^Physical Education Postdoctoral Research Station, Hebei Normal University, Shijiazhuang, China

**Keywords:** athlete, mediating role, mind wandering, personality, trait anxiety

## Abstract

**Objective:**

Mind wandering is a common phenomenon among athletes during training and competition, and can lead to poor performance. We attempt to clarify which personality type is more prone to mind wandering and the role of trait anxiety between them.

**Methods:**

Six hundred and eighty-one athletes participated in this cross sectional study. Participants completed the Athlete Mind Wandering Scale, The Chinese adjectives scale of Big-Five factor personality short scale version and Pre-Competition Emotion Scale-Trait questionnaires. The survey data was tested for common method biases, Pearson correlation analysis, and structural equation model by SPSS 25.0 and Mplus 7.0.

**Results:**

Common method biases can be accepted in this study. (1) Athletes' neuroticism was significantly and positively correlated with trait anxiety and mind wandering, respectively, athletes' extraversion, agreeableness, conscientiousness, and openness were significantly and negatively correlated with trait anxiety and mind wandering respectively; the athletes' trait anxiety was significantly and positively correlated with mind wandering; (2) By constructing mediating models, the direct effects of athletes' extraversion, agreeableness, conscientiousness, neuroticism, and openness on mind wandering were insignificant. The mediating effect of athletes' trait anxiety between the five personalities and mind wandering was significant.

**Conclusion:**

Trait anxiety in athletes plays a fully mediating role between the relationship of personality and mind wandering. Athletes' extraversion, agreeableness, conscientiousness, neuroticism, and openness can all have an impact on mind wandering through the mediating role of trait anxiety. Athletes can use the mediating role of trait anxiety to intervene the frequency of mind wandering.

## 1 Introduction

It is extremely common for athletes to experience mind wandering in training and competition. There are many cases of athletes who fail in the competitions due to wandering minds. For example, at the Tokyo 2020 Olympics, Ukraine's Sergey Kulish Shot the bullet into another competitor's target. After the match, he recalled that he felt a little uncomfortable in his clothes at the final moments of the match. This caused his mind to wander (Sohu, 2021). In the 2021 NBA preseason game, the Warriors against the Lakers. During one of the third quarter possessions, the Warriors played a pick and roll, and at that point Russell Westbrook experienced mind wandering. He did not pay attention to the defense, and when the Warriors player received the ball and prepared to attack, Russell Westbrook lunged, but the opponent hit an easy mid-range shot (Sina, 2021).

Mind wandering is a situation in which executive control shifts away from a primary task to the processing of personal goals, individuals lack control in this process (Smallwood and Schooler, 2006), and the contents of experience arise from intrinsic changes that occur within individuals (Smallwood and Schooler, 2015). Some studies detailed mind wandering to subtypes, which comprise deliberate mind wandering and spontaneous mind wandering. According to Seli et al. (2016), deliberate mind wandering is the mind wandering generated intentionally by individuals, while spontaneous mind wandering is generated unintentionally by individuals. This phenomenon that occupies roughly 30% of people's waking hours (Kane et al., 2007). Mind wandering is an extremely common phenomenon, and most athletes report experiencing mind wandering in sports (Latinjak, 2018). However, the athletes' mind wandering differs from other communities in that it often occurs during training and competition (Li, 2017). The athletes' internal information processing in this state will be separated from the current sports task, which reduces the athletes' sensitivity to the external sports information. The attention resources will be biased to the internal information of individuals, which leads to the decline of their sports performance. As the aforementioned cases of Sergey Kulish and Russell Westbrook, the occurrence of the athletes' mind wandering can have a serious negative impact on athletic performance. Researchers have interviewed athletes about the effects of experiencing mind wandering and the results have shown that mind wandering have negative even devastating effects on athletes, such as emotional fluctuation, poor training effect, decreased performance, energy consumption, and so on (Li and Yao, 2016).

Because mind wandering originates from intra-individual and it is endogenous (Song and Tang, 2012), this has led to consider personality as one of the causes of mind wandering. Personality can support the psychological selection of sports talents (Wang et al., 2016). The Big Five personality is a widely recognized personality classification, it includes following five types: extraversion, agreeableness, conscientiousness, neuroticism and openness (Costa and McCrae, 1992). Personality and mind wandering are correlated. The study with residents of Switzerland and Germany has been shown that openness is positively correlated with deliberate mind wandering (Martarelli et al., 2020). The study on university students have shown similar results: openness is positively correlated with deliberate mind wandering. Moreover, neuroticism was positively correlated with spontaneous mind wandering, while extraversion and agreeableness were negatively correlated with spontaneous mind wandering, conscientiousness was negatively correlated with both spontaneous and deliberate mind wandering (Carciofo and Jiang, 2021). Personality can directly or indirectly affect mind wandering.

Researchers have found that neuroticism can have a direct positive effect on mind wandering and conscientiousness can have a direct negative effect on mind wandering among participants from Germany, Austria, and Switzerland (Müller et al., 2021). However, another study showed that neuroticism and openness can have an indirect effect on the frequency of individual self-perceived mind wandering through meta-awareness. Moreover, neuroticism can directly and positively influence self-perceived mind-wandering frequency (Ibaceta and Madrid, 2021). Some researchers notably differentiated the situations in which mind wandering occurs. Neuroticism of the college students predicted the frequency of mind wandering in the laboratory; whereas openness predicted the frequency of mind wandering in daily life (Kane et al., 2017).

“Anxiety is a compound emotion consisting of fear, guilt, pain, and anger” (Peng and Chen, 2019), which belongs to negative emotion. Researchers classified anxiety as trait anxiety and state anxiety (Spielberger et al., 1970). Trait anxiety is a type of stable emotion and state anxiety is a kind of transient emotion. Personality is an indispensable variable when exploring anxiety. As Kotov et al. (2010) have mentioned, “a model of anxiety that does not take personality into account cannot be complete”. Studies in sports have argued that the main source of athletes' anxiety need to consider the role of personality (Wang et al., 2020). Some studies presented practical evidence of the close relationship between anxiety and personality. For example, an investigative study in China determined that extraversion, agreeableness, conscientiousness and openness were negatively correlated with anxiety, while neuroticism was positively correlated with anxiety (Tao et al., 2022). In addition, some studies have distinguished subtypes of anxiety and explored the relationship between personality and trait anxiety. For example, the participants were college students showed that neuroticism was positively correlated with trait anxiety (Robison et al., 2020) and that participants with high schizotypal personality had higher levels of trait anxiety than those with low schizotypal personality (Zhang et al., 2022). Thus, personality can influence the individuals' trait anxiety.

In addition, anxiety is closely related to mind wandering, which can increase the frequency of mind wandering (Wang, 2011). An experimental study of psychology undergraduates found that anxious individuals are more likely to experience mind wandering (Smallwood et al., 2007); The results from a clinical intervention study showed a decrease in mind wandering was associated with the improvement in anxiety (Takahashi et al., 2020); Moreover, a study on adolescents showed a positive correlation between anxiety and mind wandering (Figueiredo et al., 2020). Furthermore, as research on anxiety and mind wandering has become more and more deeper, some researchers have refined anxiety to subtypes and shifted the focus to the relationship between trait anxiety and mind wandering. Results of a study for Chinese college students showed that trait anxiety can have a direct positive effect on the frequency of mind wandering (Zhang et al., 2022). In the field of sport, anxiety has important research value. It is a negative emotion that athletes often experience during training and competition. Anxiety is considered to be the “public enemy” of athletes, which can prevent them from performing at their normal level (Lyu et al., 2018). Research has found that high levels of anxiety state can increase the frequency of mind wandering in college athletes (Wang, 2022). In our previous study, the “athlete mind wandering process model” simultaneously showed that the more negative of athletes' emotion is, the more frequency of mind wandering will occur (Li and Yao, 2016). Trait anxiety is a type of negative emotion. Combination with this theory, the higher trait anxiety of athletes, the higher frequency of mind wandering occurs.

In summary, personality can affect mind wandering and trait anxiety, respectively. Moreover, trait anxiety can affect mind wandering. Does trait anxiety play a mediating role in the relationship between personality and mind wandering? The I-PACE (Interaction of Person-Affect-Cognition-Execution) model provides circumstantial evidence for our speculations. The I-PACE model summarized the mechanisms underlying the development and maintenance of specific Internet-use disorders (Brand et al., 2016). Applying the I-PACE model to the field of sports, the order of some of the factors in this model fit perfectly into the logical structure of our speculations. The “person” in this model corresponding to our “personality”, the “affect” in this model corresponding to our “trait anxiety”, the “cognition” in this model corresponding to our “mind wandering”. In addition, our speculations has been supported by only one study, which suggested that schizotypal personality in Chinese college students could have an indirect effect on mind wandering through the mediating role of trait anxiety (Zhang et al., 2022).

In summary, it can be seen that previous studies have focused on the relationship between neuroticism, openness and mind wandering, but have not comprehensively studied the relationship between all five personalities and mind wandering. Moreover, only one type of personality was explored in previous study in relation to trait anxiety and mind wandering. The relationship between various types of personalities, trait anxiety and mind wandering has not been clarified. Finally, previous studies involved situations including daily life and laboratory, but did not involve the sports field. Mind wandering in athletes occurs in sports rather than in daily life. Thus, the relationship between personality, trait anxiety, and mind wandering may vary depending on the situation in which it occurs. This study aims to discuss the relationship between Big Five personality, trait anxiety, and mind wandering of athletes in sports situations. To form new ideas or methods to address the intervention of athletes' mind wandering. At the same time, it can provide theoretical guidance for the psychological selection of sports talents. From what has been discussed above, we proposed hypothesis: The athletes' personality can directly affect mind wandering; at the same time, the athletes' trait anxiety could mediate the relationship between personality and mind wandering.

## 2 Methods

### 2.1 Participants

Convenience sampling was used in the study, which comprises professional sports training teams in China, including the province of Hebei, Henan, Zhejiang, Sichuan, Heilongjiang, Shanghai, Beijing and so on. Questionnaires were distributed in the form of online and paper questionnaires. All participants must have at least 1 year of sports experience and have participated in competitive events. Finally, 1,245 questionnaires were collected. Six hundred and eighty-one valid questionnaires were obtained by setting two polygraph questions and checking regular responses to remove invalid questionnaires, with an efficiency rate of 54.70%. Among them, there were 350 male athletes and 331 female athletes; 262 athletes in the physique dominated event group and 419 athletes in the skill dominated event group; 315 athletes at level-2 or above and 366 athletes below the level-2; the average age was 19.44 ± 5.44 years and the average years of sports experience was 6.17 ± 4.92 years. They were informed that the participation was voluntary and anonymous. This study has been approved by the Science and Technology Ethics Committee of Hebei Normal University (NO.2023LLSC031).

### 2.2 Measurement

#### 2.2.1 Athlete mind wandering scale

The occurrence of mind wandering in competitions and training was measured using the Athlete Mind Wandering Scale, which contains five dimensions: weak attentional control, spontaneous thinking, psychological gap, competition mood, and somatic sensation, with a total of 21 items. For example, “During a competition, when I feel tired, I will be mind wandering”. All items were measured on a five-point scale (Li and Yao, 2017).

#### 2.2.2 The Chinese adjectives scale of Big-Five factor personality short scale version

The Chinese adjectives scale of Big-Five factor personality short scale version was used to measure the personality of athletes, which contains five dimensions: extraversion, agreeableness, conscientiousness, neuroticism, and openness, with a total of 20 items. For example, “worried”, “happy”. All items were measured on a six-point scale (Luo and Dai, 2018).

#### 2.2.3 Pre-competition Emotion Scale-Trait

The short-form of the Pre-competition Emotion Scale-Trait was used to measure the trait anxiety of athletes, which contains four dimensions: individual failure anxiety, social expectancy anxiety, somatic anxiety, and trait confidence, with a total of 16 items. For example, “I feel uneasy”. All items were measured on a five-point scale (Zhang, 2000).

### 2.3 Background and procedures

Data for the survey were collected from April to May 2022 using a combination of online and offline methods. Offline data collection was carried out by researchers through on-site surveys at sports training teams, where we distributed paper questionnaires. Online data were collected using the secure online survey platform “Wenjuanxing” (https://www.wjx.cn). Prior to data collection, athletes were informed that their participation was entirely voluntary, and they could opt out at any time.

### 2.4 Statistical analysis

Data obtained from the questionnaires were conducted to common method biases and correlation analysis using SPSS 25.0. Structural equation modeling was constructed using Mplus 7.0 to test for composite reliability, convergence validity, and mediating effects. The athletes' gender, age, sport level, years of experience and dominant group of the sport were added as covariants in the construction of the model to ensure further accuracy. To verify whether there is a mediating effect of trait anxiety between five personalities and mind wandering, we referred to Beyond Baron and Kenny's idea of mediation model construct (Hayes, 2009) in constructing the mediation model. We also estimated 95% confidence interval for the mediation effect by Percentile Bootstrap method and Bias-corrected BC-Bootstrap method by taking 1,000 samples, respectively. If the interval does not include zero values, it indicates that there exists significant mediating effect.

## 3 Results

### 3.1 Common method biases test

This study used the Harman method for the common method biases test of the factors to verify whether the survey had significant systematic errors (Podsakoff et al., 2003). The results of the analysis showed that a total of eight of the 51 factors had characteristic roots >1 and the first factor had an explainable percentage of 24.16%, a value lower than 40%. Results indicate that no significant common method biases exist for the factors used in this study.

### 3.2 Reliability and validity analysis

To test the reliability of each item and the ability of the dimension to explain the items, the compositional reliability and convergent validity were calculated in this study, as shown in Table 1. The composite reliability (CR) of each dimension was above the recommended level of 0.7. The average variance extracted (AVE) is the average explanatory power of the dimensions for the items. All dimensions' AVE was >0.36, indicating that they were within the acceptable range and most of them conformed to the idealized criteria of 0.5.

**Table 1:** Reliability and convergent validity analysis.

**Dim**.	**Item**	**Parameters of significant test**				**Item Reliability**	**Composite Reliability**	**Convergence Validity**
		**Estimate**	**S.E**.	**Est./S.E**.	***P*-value**	**SMC**	**CR**	**AVE**
WAC	MW1	0.600	0.029	21.002	^***^	0.360	0.714	0.459
	MW17	0.803	0.021	38.476	^***^	0.645		
	MW14	0.611	0.028	22.099	^***^	0.373		
ST	MW12	0.606	0.027	22.356	^***^	0.367	0.770	0.458
	MW16	0.711	0.023	31.604	^***^	0.506		
	MW19	0.631	0.026	24.370	^***^	0.398		
	MW21	0.748	0.021	35.558	^***^	0.560		
PG	MW3	0.669	0.025	26.636	^***^	0.448	0.774	0.534
	MW8	0.761	0.021	36.469	^***^	0.579		
	MW13	0.759	0.021	36.526	^***^	0.576		
CM	MW6	0.660	0.026	24.966	^***^	0.436	0.779	0.541
	MW9	0.786	0.021	36.979	^***^	0.618		
	MW11	0.755	0.022	33.747	^***^	0.570		
SS	MW10	0.669	0.024	27.682	^***^	0.448	0.843	0.574
	MW15	0.809	0.017	47.687	^***^	0.654		
	MW18	0.775	0.019	41.548	^***^	0.601		
	MW20	0.770	0.019	40.744	^***^	0.593		
IFA	A3	0.770	0.020	38.828	^***^	0.593	0.807	0.582
	A7	0.736	0.022	34.205	^***^	0.542		
	A11	0.782	0.019	40.453	^***^	0.612		
SEA	A4	0.788	0.020	39.793	^***^	0.621	0.806	0.581
	A8	0.753	0.021	35.302	^***^	0.567		
	A16	0.746	0.021	34.805	^***^	0.557		
SA	A2	0.636	0.027	23.971	^***^	0.404	0.819	0.532
	A6	0.751	0.021	35.764	^***^	0.564		
	A14	0.731	0.022	33.258	^***^	0.534		
	A17	0.791	0.019	41.821	^***^	0.626		
TC	A1	0.728	0.023	31.937	^***^	0.530	0.831	0.556
	A5	0.761	0.021	35.517	^***^	0.579		
	A9	0.614	0.028	22.279	^***^	0.377		
	A13	0.858	0.018	47.768	^***^	0.736		
E	P1	0.575	0.029	19.882	^***^	0.331	0.842	0.576
	P6	0.737	0.021	34.878	^***^	0.543		
	P11	0.871	0.016	53.875	^***^	0.759		
	P16	0.819	0.018	45.245	^***^	0.671		
A	P2	0.617	0.030	20.801	^***^	0.381	0.789	0.485
	P7	0.757	0.025	30.196	^***^	0.573		
	P12	0.692	0.027	25.702	^***^	0.479		
	P17	0.712	0.026	26.955	^***^	0.507		
C	P3	0.601	0.031	19.272	^***^	0.361	0.763	0.449
	P8	0.748	0.026	28.241	^***^	0.560		
	P13	0.746	0.027	28.118	^***^	0.557		
	P18	0.566	0.032	17.454	^***^	0.320		
N	P4	0.610	0.029	21.142	^***^	0.372	0.813	0.524
	P9	0.789	0.022	35.993	^***^	0.623		
	P14	0.751	0.023	32.153	^***^	0.564		
	P19	0.732	0.024	30.918	^***^	0.536		
O	P5	0.586	0.031	18.892	^***^	0.343	0.748	0.435
	P10	0.823	0.027	30.074	^***^	0.677		
	P15	0.688	0.030	23.049	^***^	0.473		
	P20	0.494	0.036	13.836	^***^	0.244		

^***^p < 0.001.

WAC, weak attentional control; ST, spontaneous thinking; PG, psychological gap; CM, competition mood; SS, somatic sensation; IFA, individual failure anxiety; SEA, Social Expectancy anxiety; SA, somatic anxiety; TC, trait confidence E, extraversion; A, agreeableness; C, conscientiousness; N, neuroticism; O, openness; SMC is the ability of the dimension to explain the items.; AVE is the average explanatory power of a dimension on the items >0.36 is acceptable.

To test the discriminant validity among the dimensions, the correlation coefficients and the square root of all AVEs between the dimensions were calculated for comparison in this study, as shown in Table 2. The square root of all AVEs were above the level of 0.5 and were basically higher than the correlation coefficients with other dimensions, which indicates that the scales had good discriminant validity.

**Table 2:** Discriminate validity of each dimension.

**Dim**.	**WAC**	**ST**	**MG**	**CM**	**SS**	**IFA**	**SEA**	**SA**	**TC**	**E**	**A**	**C**	**N**	**O**
WAC	**0.677**													
ST	0.669	**0.677**												
PG	0.633	0.659	**0.731**											
CM	0.523	0.612	0.600	**0.736**										
SS	0.653	0.700	0.648	0.565	**0.758**									
IFA	0.328	0.375	0.282	0.390	0.289	**0.763**								
SEA	0.332	0.422	0.312	0.339	0.349	0.696	**0.762**							
SA	0.316	0.404	0.310	0.352	0.357	0.615	0.654	**0.729**						
TC	−0.189	−0.177	−0.165	−0.275	−0.135	−0.340	−0.202	−0.234	**0.746**					
E	−0.136	−0.097	−0.110	−0.127	−0.101	−0.164	−0.129	−0.159	0.312	**0.759**				
A	−0.161	−0.099	−0.122	−0.134	−0.063	−0.188	−0.141	−0.185	0.274	0.720	**0.696**			
C	−0.204	−0.182	−0.144	−0.182	−0.130	−0.266	−0.172	−0.233	0.321	0.528	0.685	**0.670**		
N	0.199	0.174	0.167	0.214	0.144	0.320	0.277	0.293	−0.370	−0.716	−0.759	−0.722	**0.724**	
O	−0.094	−0.115	−0.117	−0.186	−0.096	−0.144	−0.056	−0.068	0.388	0.549	0.470	0.466	−0.559	**0.660**

The diagonal bold text is the square root of all AVEs ($\sqrt{AVE}$), and the lower triangular value is the Pearson correlation coefficient of dimensions. WAC, weak attentional control; ST, spontaneous thinking; PG, psychological gap; CM, competition mood; SS, somatic sensation; IFA, individual failure anxiety; SEA, social expectancy anxiety; SA, somatic anxiety; TC, trait confidence; E, extraversion; A, agreeableness; C, conscientiousness; N, neuroticism; O, openness.

### 3.3 Correlation among personality, mind wandering, and trait anxiety in athletes

To explore the relationship between each two variables separately, data was analyzed using Pearson correlation. The correlation analysis results between the variables, and the correlation coefficients *r* between the variables were all at a significant level (Table 3). The athletes' neuroticism was significantly and positively correlated with mind wandering (*r* = 0.214, *p* < 0.01), athletes' extraversion, agreeableness, conscientiousness and openness were all significantly and negatively correlated with mind wandering (*r* = −0.136 to −0.201, *p* < 0.01); athletes' neuroticism was significantly and positively correlated with trait anxiety (*r* = 0.404, *p* < 0.01), athletes' extraversion, agreeableness, conscientiousness and openness were all significantly and negatively correlated with trait anxiety (*r* = −0.204 to −0.315, *p* < 0.01); the athletes' trait anxiety was significantly and positively correlated with mind wandering (*r* = 0.475, *p* < 0.01).

**Table 3:** Descriptive statistics and correlation analysis of mind wandering, personality and trait anxiety.

	**M**	**SD**	**1**	**2**	**3**	**4**	**5**	**6**	**7**
1	1.260	0.391	1.000						
2	2.064	0.601	−0.136^**^	1.000					
3	2.203	0.547	−0.137^**^	0.720^**^	1.000				
4	2.071	0.518	−0.201^**^	0.528^**^	0.685^**^	1.000			
5	1.463	0.551	0.214^**^	−0.716^**^	−0.759^**^	−0.722^**^	1.000		
6	1.939	0.519	−0.146^**^	0.549^**^	0.470^**^	0.466^**^	−0.559^**^	1.000	
7	1.509	0.469	0.475^**^	−0.241^**^	−0.250^**^	−0.315^**^	0.404^**^	−0.204^**^	1.000

^**^p < 0.01 (two-tailed). 1, mind wandering; 2, extraversion; 3, agreeableness; 4, conscientiousness; 5, neuroticism; 6, openness; 7, trait anxiety.

### 3.4 Mediating effect of trait anxiety between personality and mind wandering in athletes

To verify the mediating effect of trait anxiety between personality and mind wandering in athletes, we used Mplus 7.0 to establish five mediation models for hypothesis testing by using the five personality types of athletes as latent variables. The personality variable in model M1 was extraversion; the personality variable in model M2 was agreeableness; the personality variable in model M3 was conscientiousness; the personality variable in model M4 was neuroticism; and the personality variable in model M5 was openness. Six items were removed from the original 57 items to achieve a standard fitness, and 51 items were finally retained (the six items were removed from all data results in this study). Due to the existence of second-order model constructs for trait anxiety and mind wandering, we conducted the confirmatory factor analysis (CFA) to compare the second-order model with the first-order model. The target coefficient was 0.9, demonstrating that the second-order model can replace the first-order model.

**Figure 1:**
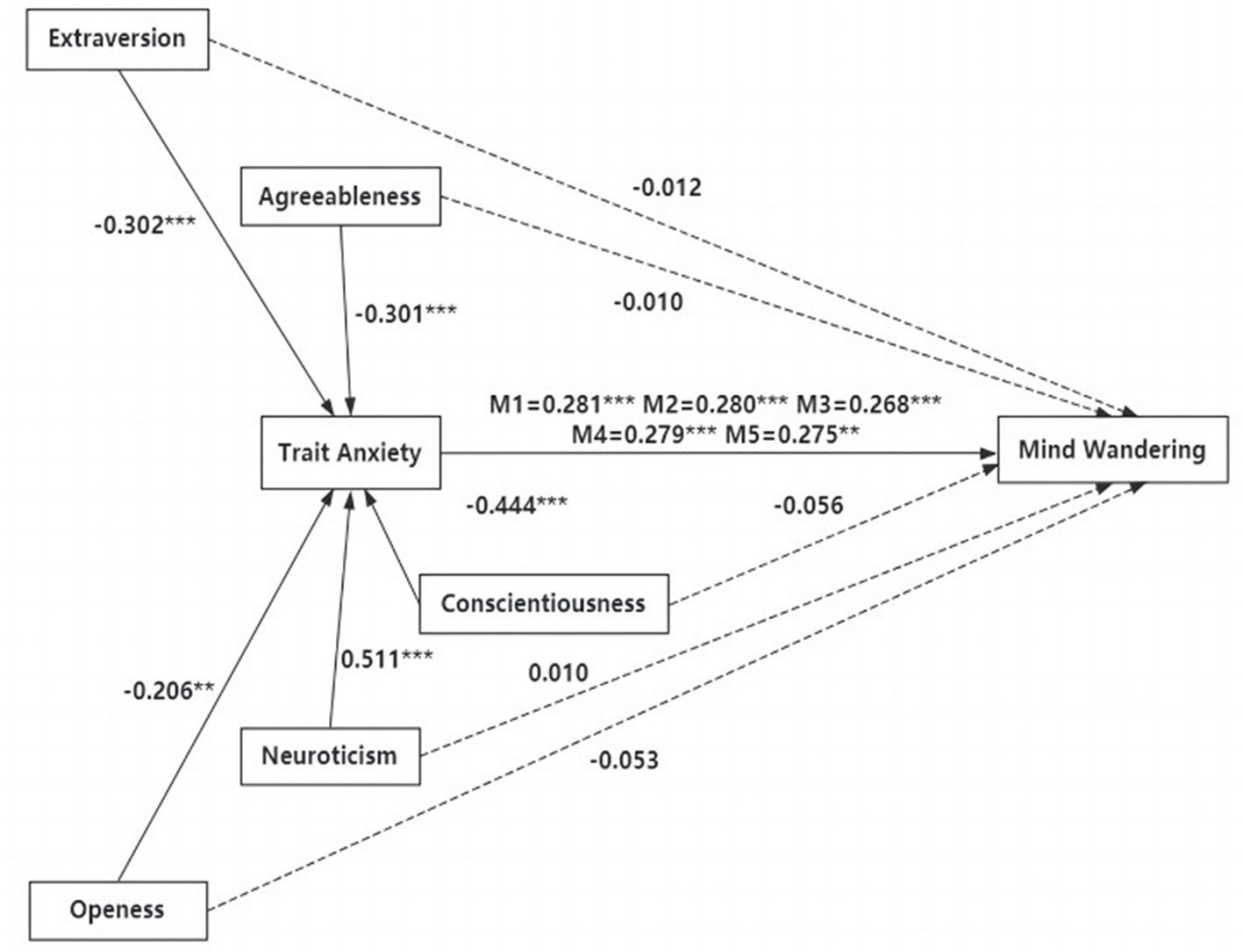
The influence paths of trait anxiety between Big Five personality and athletes' mind wandering.

The goodness of fit indices and path effects of the five mediating models were shown in Tables 4, 5, while the models' path coefficients were shown in Figure 1. Table 4 showed that these statistical values indicate that the structural models fit well. According to the results of the mediation effect test in Table 5, the total effects of models M1, M2, M3, M4, and M5 were significant. The direct effects of the five mediating models showed that none of the direct effects of athletes' extraversion, agreeableness, conscientiousness, neuroticism, and openness on mind wandering were significant (β = −0.056 to 0.010, *p* = 0.076–0.742). The five models showed significant mediating effects of athletes' trait anxiety between extraversion, agreeableness, conscientiousness, neuroticism, openness and mind wandering (β = −0.119 to 0.143, *p* = 0.000–0.006). Thus, all five mediation models validate that trait anxiety in the athlete population plays a fully mediating role between personality and mind wandering.

**Table 4:** Fitness index of each mediating model.

**Model**	**χ^2^**	**χ^2^/df**	**CFI**	**TLI**	**RMSEA**	**SRMR**
	More smaller more better	1 < χ ^2^/df < 3	>0.900	>0.900	< 0.08	< 0.08
M1	1,623.446	2.293	0.921	0.914	0.044	0.050
M2	1,589.139	2.245	0.921	0.914	0.043	0.047
M3	1,624.490	2.294	0.918	0.911	0.044	0.049
M4	1,622.437	2.292	0.920	0.913	0.044	0.050
M5	1,683.328	2.378	0.912	0.905	0.045	0.054

M1, extraversion; M2, agreeableness; M3, conscientiousness; M4, neuroticism; M5, openness.

**Table 5:** Bootstrap and BC-Bootstrap analysis of mediating effect test of trait anxiety.

**Path**	**Estimate**	**S.E**.	**Est./S.E**.	***p-*value**	**Bootstrap**		**BC-Bootstrap**	
					**Lower**	**Upper**	**Lower**	**Upper**
M1T	−0.096	0.034	−2.871	0.004	−0.162	−0.033	−0.163	−0.034
M1ID	−0.085	0.020	−4.317	0.000	−0.125	−0.051	−0.126	−0.051
M1D	−0.012	0.030	−0.394	0.693	−0.069	0.049	−0.068	0.052
M2T	−0.095	0.031	−3.042	0.002	−0.160	−0.040	−0.161	−0.040
M2ID	−0.085	0.018	−4.579	0.000	−0.122	−0.051	−0.126	−0.051
M2D	−0.010	0.027	−0.375	0.708	−0.064	0.043	−0.064	0.045
M3T	−0.175	0.041	−4.311	0.000	−0.266	−0.102	−0.266	−0.102
M3ID	−0.119	0.024	−4.856	0.000	−0.175	−0.077	−0.180	−0.078
M3D	−0.056	0.033	−1.711	0.087	−0.123	0.006	−0.121	0.007
M4T	0.153	0.032	4.723	0.000	0.095	0.221	0.098	0.225
M4ID	0.143	0.023	6.183	0.000	0.101	0.194	0.104	0.200
M4D	0.010	0.032	0.329	0.742	−0.052	0.071	−0.052	0.071
M5T	−0.110	0.034	−3.284	0.001	−0.176	−0.044	−0.180	−0.052
M5ID	−0.057	0.021	−2.767	0.006	−0.098	−0.019	−0.099	−0.020
M5D	−0.053	0.030	−1.777	0.076	−0.110	0.008	−0.109	0.009

“T” represents total effect, “ID” represents indirect effect, and “D” represents direct effect; upper and lower limits are the lower and upper limits of the 95% confidence interval.

## 4 Discussion

To explore the influencing factors of athletes' mind wandering, this study verified the relationship between the five personalities, trait anxiety, and mind wandering through structural equation modeling. Surprisingly, trait anxiety played a fully mediating role between the effects of all five personalities on mind wandering. All five personalities of the athletes had an indirect effect on mind wandering through trait anxiety.

### 4.1 The relationship between personality and athletes' mind wandering in the mediation model

The correlation analysis showed that the athletes' neuroticism was positively correlated to mind wandering, while extraversion, agreeableness, conscientiousness and openness were negatively correlated with mind wandering. Carciofo and Jiang (2021) divided mind wandering to deliberate and spontaneous mind wandering, and found that Chinese college students' neuroticism was positively correlated with spontaneous mind wandering, extraversion and agreeableness were negatively correlated with spontaneous mind wandering, and conscientiousness was negatively correlated with both spontaneous and deliberate mind wandering, and the results of this study were similar to our findings. The direction of correlation between neuroticism and mind wandering is different from the other four personalities. Neuroticism causes individuals to tend to focus on their inner world and can positively affect individuals' meta-awareness (Ibaceta and Madrid, 2021). That is, individuals with neuroticism have a greater meta-awareness of mind wandering, which may contribute to the higher frequency of mind wandering reported by this type of personality. Therefore, there is a positive correlation between neuroticism and mind wandering in athletes. However, the difference is that previous studies have found a positive correlation between openness and deliberate mind wandering in resident groups from Switzerland and Germany (Martarelli et al., 2020). Our study found inconsistent result: a negative correlation between openness and mind wandering in athletes. There are two reasons for the discrepancy between the results of our study and the previous studies. First, the previous studies distinguished between spontaneous and deliberate mind wandering, while our study did not classified subtypes of mind wandering. Second, the mind wandering measured in this study occurred in sports, which is a large situational difference from mind wandering in daily life.

Surprisingly, dramatic results emerged after adding the athletes' trait anxiety as a mediating variable in the models. Results showed that none of the direct effects between the athletes' five personalities and mind wandering were significant. The athletes' personality did not have a direct effect on mind wandering. In contrast, a previous study on the college student population showed a significant direct effect between neuroticism and self-perceived frequency of mind wandering (Ibaceta and Madrid, 2021). Our result appears difference from the result of previous study and also partially disproves our hypothesis. First, a previous research has found that there were some differences between athletes' Big Five personality and non-athletes'. Researchers explained the reasons for this difference: sports and exercise activity are elective contexts of expression and development of energy features in individuals (Steca et al., 2018). Second, mind wandering in this study belong to sports situations, which differ from daily life situations. These reasons may reduce the direct effect of personality on mind wandering in the athletes.

Thus, the direct effect of personality on mind wandering may have situational difference, and the results of studies conducted for daily life cannot be applied to sports straightly. The result of this study contributes significantly to the validation of the relationship between personality and mind wandering in sports. Our result shows that if the psychological selection of sports talents is conducted from the perspective of mind wandering, the personality cannot be considered as the only indicator. This also indicates to researchers that the situational specificity should not be overlooked when exploring the relationship between mind wandering and other variables in the future. Future studys can search for other variables as psychological selection indicators for judging athletes' attentional quality.

### 4.2 Mediating role of trait anxiety between personality and mind wandering in athletes

We found that the five personalities of athletes exhibited different trends of correlation with trait anxiety in the correlation analysis. Athletes' extraversion, agreeableness, conscientiousness, and openness were all negatively correlated with trait anxiety, while neuroticism was positively correlated with trait anxiety. This result was consistent with the findings of a study, which found that college students' neuroticism was positively correlated with trait anxiety (Robison et al., 2020). Highly neurotic individuals had more intense emotional reactions (Huang et al., 2015). Individuals with high neuroticism may be more sensitive and “neuroticism characterized by emotional susceptibility, impulsivity, anxiety, and escapism” (Zhang et al., 2021). This possibly leads to a positive correlation between neuroticism and trait anxiety in athletes. By contrast, the other four personalities of athletes were all negatively correlated with trait anxiety. This is similar to the result of a study in which the participants were Chinese firefighters. That study found that extraversion, agreeableness, conscientiousness, and openness were all negatively correlated with anxiety (Tao et al., 2022). Due to extraversion exhibits optimism; agreeableness exhibits straightforward; conscientiousness exhibits self-discipline and openness exhibits creative (Peng and Chen, 2019). Therefore, we extrapolate that the four types of personalities have more positive overtones compared to the neuroticism. This may lead to a negative correlation between extraversion, agreeableness, conscientiousness, openness, and trait anxiety in athletes.

In addition, trait anxiety in athletes was positively correlated with mind wandering. This result is similar to the previous study (Zhang et al., 2022), which found that trait anxiety was positively correlated with mind wandering in Chinese young adults. Our study also confirmed the theoretical model of the “athlete mind wandering process model”, which suggests that the more negative of athletes' emotion is, the more frequency of mind wandering will occur (Li and Yao, 2016). Athletes may often consider the “desire to win or fear losing” in competition, which leads to a high level of trait anxiety over time. This can lead athletes to focus more of their attention on internal thoughts, resulting in a higher frequency of mind wandering. Furthermore, the athlete in interviews from qualitative studies mentioned that mind wandering occurs because of anxiety (Li and Yao, 2016).

The results of the mediating effects in this study showed that the model with neuroticism as a personality variable (M4) showed a different trend of effect from the other four models. In model M4, the athletes' neuroticism positively affects trait anxiety, whereas in the other models, extraversion, agreeableness, conscientiousness, and openness all negatively affect trait anxiety. In conclusion, all five personalities of athletes can indirectly influence mind wandering through trait anxiety. This result supports Hypothesis II. Zhang et al. (2022) found that schizotypal personality in the college student population can have an indirect effect on mind wandering through the mediating role of trait anxiety. This result is similar to the results of our study. Both results highlighted that trait anxiety can play an important role between personality and mind wandering.

The M4 mediating effect showed that the neuroticism of athletes can have an indirect effect on mind wandering by increasing the level of trait anxiety. Why do athletes' neuroticism can increase the level of trait anxiety? First, neuroticism is considered to be the most powerful anxiety-prone trait (Zhang and An, 2016). A study showed that the college students high in neuroticism reported feeling more anxiety (Carciofo and Jiang, 2021), and neuroticism can have a direct and positive affect on trait anxiety (Su and Wang, 2014). Second, because neuroticism exhibits characteristics such as being prone to be emotional (Zhang et al., 2021). Then athletes with high neuroticism are prone to be emotional when faced with rapidly changing sports situations during training and competition, which may lead to their level of trait anxiety increased. This supports the result that athletes' neuroticism can directly and positively affect trait anxiety. Furthermore, the positive effect of trait anxiety on mind wandering corroborates the theoretical model of the “athlete mind wandering process model” (Li and Yao, 2016). That is, the more negative of athletes' emotion is, the more frequency of mind wandering will occur. Thus, the mediating effect of trait anxiety in athletes between neuroticism personality and mind wandering is more logical. This result suggests that coaches can guide athletes with high neuroticism to reduce the level of trait anxiety, and then the occurrence of mind wandering and its negative effects on athletes can be indirectly reduced.

However, results of models M1, M2, M3, and M5 showed that the athletes' extraversion, agreeableness, conscientiousness and openness all have indirect effect on mind wandering by reducing the level of trait anxiety. It is clear that the model path trends for M1, M2, M3, and M5 different from that of M4. Why are there differences in the paths of influence of these four personalities on trait anxiety compared to neuroticism? First, referring to previous study, personality should be considered as the influencing factor on anxiety (Kotov et al., 2010). Personality have the most direct effect on trait anxiety. We have mentioned above that the other four types of personalities have more positive overtones compared to the neuroticism. In addition, the modulation of anxiety by athletes with different personality may also make a difference in the path of influence between the two. Research in sports has shown that good freestyle aerialists are all extroverted personality types. They are better able to regulate their emotion and respond in a timely and positive manner to reduce negative anxiety states (Pan, 2019). Among the Big Five personality, extraversion, agreeableness, conscientiousness, and openness are all positive and extroverted personalities compared to neuroticism. Thus, these four personalities may negatively influence trait anxiety in athletes. This result suggests that we should favor athletes with extraversion, agreeableness, conscientiousness and openness when selecting them in practice. Avoiding them to damage their performance during training and competition due to their higher level of trait anxiety.

How do athletes' extraversion, agreeableness, conscientiousness, and openness reduce trait anxiety respectively? First, extraversion is characterized by cheerfulness and optimism (Zhou et al., 2000), so athletes with high extraversion tend to maintain an positive and aggressive attitude and suppress their negative emotions, thus avoiding higher trait anxiety. Second, agreeableness exhibits altruistic (Zhou et al., 2000), and athletes with agreeableness may be able to do well in teamwork, thus enabling them to gain more team belonging in the face of training or competition and moderating the generation of trait anxiety. Third, conscientiousness is characterized by orderliness and effort (Zhou et al., 2000). Athletes with high conscientiousness tend to make scientific and reasonable arrangements for their daily training plans, and implementing the plans in a step-by-step manner enables them not to have a higher level of trait anxiety in training or competition. Finally, openness is characterized by flexible thinking, innovative and imaginative (Zhou et al., 2000), it is positively correlated with creative thinking (Li et al., 2022). Highly openness athletes may create more unique movements or combinations of techniques and tactics during training and competition. Their innovative tactical or movement combinations may steadily improve their performance. Over the long term, they may be in a chronically positive mood, thus contributing to lower level of trait anxiety in themselves. Based on the negative effect of these four personalities on trait anxiety, the path of the model that trait anxiety positively affects mind wandering confirmed the theoretical model of our previous study. According to “Athlete mind wandering process model” (Li and Yao, 2016), the more negative of athletes' emotion is, the more frequency of mind wandering will occur. Therefore, the mediating role of athletes' trait anxiety between the Big Five personality and mind wandering has been justified in theory and practice.

The results of the study have important implications for the development of the theory: (1)There are two limitations of previous studies. First, previous studies only explored the relationship among one certain personality type, trait anxiety and mind wandering. Second, previous studies were limited to daily life situation, and the results could not be applied to sports. To bypass the above two limitations, this study specifically focused on sports situation, verified the relationship among all the five personality types, trait anxiety and mind wandering. (2) The results of this study validated the theoretical model of the “athlete mind wandering process model” constructed by our research team in previous studies (Li and Yao, 2016). This study can make an outstanding contribution to the series of studies on “athlete mind wandering”.

## 5 Limitations and future directions

In fact, some limitations exist in this study. First, this study used a self-report method to measure mind wandering, and the measurement instrument was an athlete's self-assessment scale rather than objective behavioral and physiological indicators. However, compared to the “probe”, retrospective questionnaires can avoid disrupting participants' natural thinking (Mis and Kowalczyk, 2020), which can ensure the integrity of thinking. Second, in the process of investigation, we selected athletes from a variety of sports. The diverse sports are more representative of the whole group characteristics of athletes, and the results of this study have more holistic characteristics of athletes. The results only have general significance for guiding athletes and lack pertinence, wherein we could not reflect the targeted characteristics of athletes in some sports.

Future studies can combine experimental method in which mind wandering can be measured objectively and the results can be more accurate. In addition, researchers can specifically explore athletes in a particular sport when conducting similar studies in the future and combine the research results with the characteristics of that sport, wherein the results are more project-specific in guiding practice. Lastly, the results of this study primarily highlighted the mediating role of trait anxiety between athletes' personality and mind wandering. Future studies could provide psychological interventions for the frequency of mind wandering by reducing individual trait anxiety.

## 6 Conclusion

This study explored the relationship between the Big Five personality, trait anxiety, and mind wandering in athletes by constructing structural equation models. It was found that athletes' trait anxiety can play a fully mediating role between personality and mind wandering. The athletes' extraversion, agreeableness, conscientiousness, neuroticism, and openness can indirectly influence mind wandering through trait anxiety. This result has important implications for guiding sports practice. First, the mediating effect of trait anxiety can be used as a warning: by reducing the anxiety level of athletes, the occurrence of mind wandering in sports situations can be intervened, in turn reducing the negative effect of mind wandering on performance. In addition, the personality cannot be considered as the only indicator during psychological selection of athlete who are rarely mind wandering. Coaches should pay attention to the impact of athletes' trait anxiety on mind wandering.

## Data availability statement

The original contributions presented in the study are included in the article/Supplementary material, further inquiries can be directed to the corresponding author.

## Ethics statement

The studies involving humans were approved by the Science and Technology Ethics Committee of Hebei Normal University. The studies were conducted in accordance with the local legislation and institutional requirements. Written informed consent for participation in this study was provided by the participants' legal guardians/next of kin.

## Author contributions

YL and JL conceived of the study, analyzed the data, and drafted manuscript. JM and YX have collected the data and revised the manuscript. All authors contributed to the article and approved the submitted version.
